# 3′,4′-Dihydroxyflavonol Inhibits Fibrotic Response in a Rabbit Model of Glaucoma Filtration Surgery

**DOI:** 10.3390/ijms251910767

**Published:** 2024-10-07

**Authors:** Zoe Pasvanis, Roy C. K. Kong, Manisha H. Shah, Elsa C. Chan, Jennifer C. Fan Gaskin

**Affiliations:** 1 Ophthalmology, Department of Surgery, University of Melbourne, Fitzroy, VIC 3065, Australia; 2 Centre for Eye Research Australia, Royal Victorian Eye and Ear Hospital, East Melbourne, VIC 3002, Australia; 3 Department of Medicine, St Vincent’s Hospital, University of Melbourne, Fitzroy, VIC 3065, Australia; 4 Glaucoma Research and Investigation Unit, Royal Victorian Eye and Ear Hospital, East Melbourne, VIC 3002, Australia

**Keywords:** antifibrotic, conjunctival scarring, glaucoma filtration surgery

## Abstract

Post-operative fibrosis of the filtering bleb limits the success of glaucoma filtration surgery (GFS). To minimise subconjunctival scarring following GFS, treatment with antimetabolites such as Mitomycin C (MMC) has become standard practice; however, their use is associated with considerable side effects. This study aimed to investigate the anti-scarring properties of 3′,4′-dihydroxyflavonol (DiOHF). GFS was performed in New Zealand white rabbits who received eye drops of DiOHF three times daily and vehicle eye drops after surgery (*n* = 5) or a single intraoperative treatment of MMC (*n* = 5). Blebs were imaged immediately following surgery and on days 7, 15, 21, and 28 for clinical examination. On day 28, eyes were harvested to assess collagen deposition, expression of α-SMA, oxidative stress, angiogenesis, fibroblast activity, and inflammation in the conjunctiva/Tenon’s layer. At 7 and 28 days post-GFS, MMC-treated blebs were more ischaemic than DiOHF- or vehicle-treated blebs. On day 28, DiOHF treatment significantly suppressed collagen accumulation, CD31 expression, Vimentin expression, and CD45 expression compared to the vehicle control. No difference was observed in 3-Nitrotyrosine or αSMA expression between treatment groups. Treatment with DiOHF reduced conjunctival scarring and angiogenesis in rabbits with GFS, which was comparable to MMC. DiOHF may be a safer and more effective wound-modulating agent than conventional antifibrotic therapy in GFS.

## 1. Introduction

Glaucoma currently affects approximately 300,000 Australians and is projected to affect a staggering 111.8 million people globally by 2040 [1,2]. As such, glaucoma has become a leading cause of irreversible blindness worldwide. Glaucoma filtration surgery (GFS) remains the most reliable surgical treatment for medically uncontrolled glaucoma [3]. The aim of GFS is to lower intraocular pressure (IOP), a key modifiable risk factor associated with this disease, in a controlled manner to protect against pressure-induced optic neuropathy. This procedure involves the creation of an alternative drainage pathway from the anterior chamber to the subconjunctival space to allow for excess aqueous humour to bypass the trabecular meshwork and drain into a subconjunctival filtering bleb. However, as a result of the injury and inflammation created by the surgical wound, the body’s natural wound-healing response is triggered. This process involves the transforming growth factor (TGFβ)-mediated activation of Tenon’s fibroblasts, located in the Tenon’s layer of the conjunctiva, into myofibroblasts. Once activated, these cells proliferate and produce excess extracellular matrix (ECM) components such as collagens and fibronectin, which contribute to excessive fibrous scar formation and subsequent bleb filtering failure [4]. Postoperative conjunctival fibrosis at the bleb has been found to be the most common cause of GFS failure [5].

In an effort to combat excessive postoperative scarring, potent chemotherapeutic antimetabolite agents, like Mitomycin C (MMC), as adjunctive antifibrotic therapy, are currently being used in clinical practice. While these agents have certainly improved the success of GFS, the failure rate of GFS at 5 years can still be as high as 50% due to scarring [6]. This is because MMC can only be applied at a low concentration for a few minutes at the surgical site to avoid toxicity. Despite the very judicious administration of these agents by clinicians, their use continues to be associated with an increased incidence of delayed hypotony from late-onset bleb leakage [7]. The non-specific cytotoxic action of these standard-of-care antimetabolites can lead to a higher risk of cataract formation, corneoscleral damage, wound dehiscence and endophthalmitis that can lead to blindness and enucleation [8,9,10]. Hence, there is an unmet clinical need for a safer and more specific antifibrotic agent for GFS.

The TGFβ-mediated activation of Tenon’s fibroblasts into myofibroblasts is driven by redox imbalance, where oxidative stress is increased [11]. The overproduction of reactive oxygen species (ROS) causing redox imbalance contributes to the pathophysiology of many profibrotic diseases [12,13]. In this way, treatment with an antioxidant agent is appealing for minimising the post-GFS wound healing response, as antioxidants neutralise free radicals. Of all known dietary antioxidants, flavonoids are the most abundant in our diet, with a variety of fruits, vegetables, wine and green tea being rich sources of flavonols (a subclass of flavonoids) [14]. 3′,4′- Dihydroxyflavonol (DiOHF) is a synthetic flavonoid that has been shown to have cardioprotective and antioxidant effects in various animal models of myocardial ischaemia/reperfusion injury [15,16]. By possessing a 3′,4′-catechol (dihydroxy) component within its chemical structure, DiOHF is equipped to be a very effective ROS scavenger [17]. In this way, we anticipate that DiOHF can effectively minimise the redox imbalance, which problematically promotes post-GFS fibrosis. Recent work within our group has revealed the inhibitory effects of DiOHF in a mouse model of GFS, where the systemic administration of DiOHF suppressed angiogenesis and conjunctival scarring [18]. Hence, we hypothesised that DiOHF will suppress postoperative fibrosis in a rabbit model of GFS. The objective of this study was to evaluate the antifibrotic effect of topical DiOHF eye drops in a preclinical rabbit model of GFS in comparison to the standard-of-care MMC treatment.

## 2. Results

### 2.1. MMC Treatment Produced Initially Ischaemic Blebs, While Other Treatments Indicated Reduced Bleb Inflammation over Time

**Figure 1:**
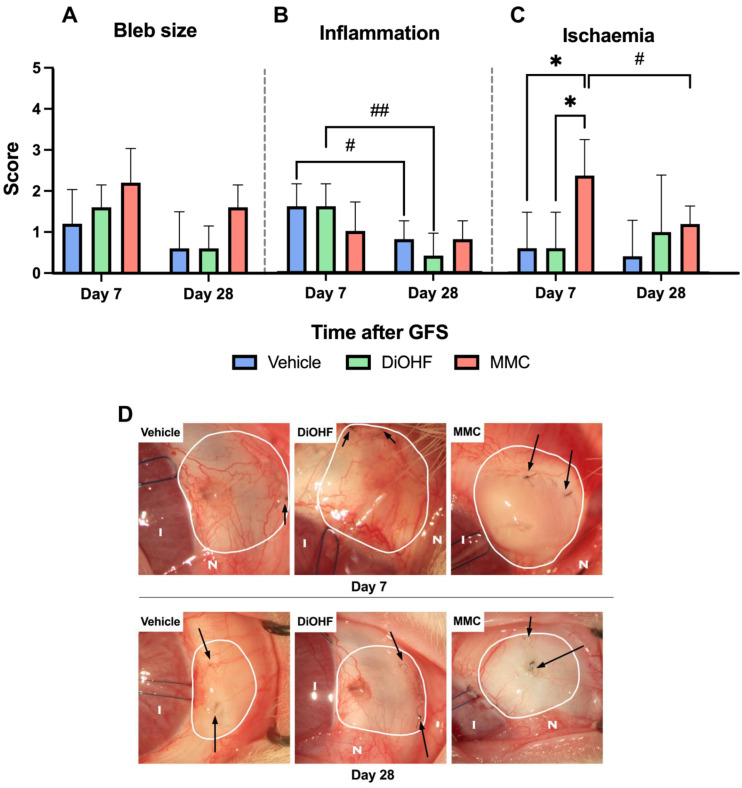
Bleb characteristics of treatment groups at 7 and 28 days after GFS. Blinded scoring of blebs treated with the vehicle DiOHF and MMC (*n* = 5) at days 7 and 28 post-GFS with regard to (**A**) bleb size, (**B**) degree of inflammation at bleb and (**C**) level of ischaemia at bleb. Scoring according to Table S1. Statistical significance between treatments within a time point is indicated by * for *p* < 0.05 (Two-way ANOVA with Šídák’s post hoc test). Statistical significance between time points is indicated by # for *p* < 0.05 and ## for *p* < 0.001 (Two-way ANOVA with Šídák’s post hoc test). (**D**) Representative days 7 and 28 macroscopic images of rabbit blebs treated with the vehicle, DiOHF and MMC. Arrows point to sutures used to close the surgical wound created during GFS. I, iris; N, nictitating eyelid (where visible).

No difference in bleb size was identified clinically in the rabbits at days 7 and 28 (Figure 1A). On day 7, blebs treated with MMC presented significantly more ischaemic blebs than those treated with the vehicle or DiOHF (Figure 1C). MMC-induced ischaemia was observed as white, non-vascular blebs (Figure 1D, Figure S1C,F,L,I,O). However, by day 28, this ischaemia lessened such that there was no significant difference between the MMC, DiOHF, and vehicle treatment groups (Figure 1C). Days 7 and 28 revealed that MMC treatment yielded no change in bleb inflammation; however, blebs in the vehicle and DiOHF treatment groups exhibited a decrease in inflammation over time (Figure 1B). These reductions in inflammation are observed in the Day 28 vehicle and DiOHF blebs, which contain fewer blood vessels and less redness than in the Day 7 vehicle and DiOHF blebs (Figure 1D). This gradual reduction in bleb redness and blood vessels is observed at Days 0, 7, 15, 21 and 28 in vehicle-treated blebs (Figure S1A,D,G,J,M) and DiOHF-treated blebs (Figure S1B,E,H,K,N).

### 2.2. DiOHF Suppressed Collagen Accumulation in Rabbits with GFS

**Figure 2:**
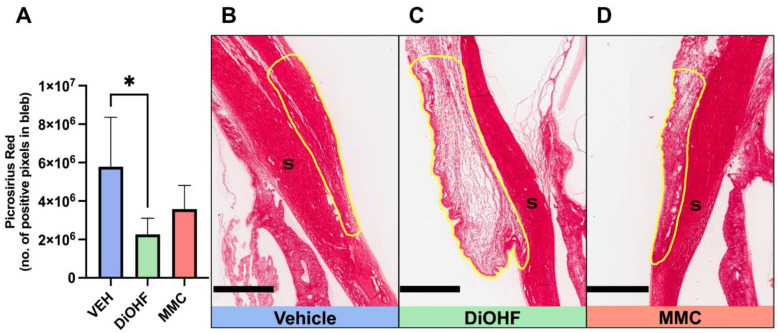
Collagen accumulation in blebs at 28 days post-GFS. (**A**) Quantitative data, by positive pixel count, of total picrosirius red-positive bleb areas following post-GFS treatment with the vehicle, DiOHF or MMC. Vehicle, 0.01% dimethyl sulfoxide (DMSO); DiOHF, 10 μM; MMC, 0.4 mg/mL (One-way ANOVA with Tukey’s post hoc test, *n* = 5, * indicates a significance of *p* < 0.05. Data are presented as mean ± SD). (**B**–**D**) Representative images of collagen accumulation at blebs, outlined in yellow, from operated rabbit eyes that received post-GFS treatment at the surgical site with either daily vehicle eye drops, daily DiOHF eye drops or a one-time intraoperative application of MMC. Picrosirius red-positive areas indicating collagen accumulation are stained red. S, sclera. Scale bar, 700 μm.

Despite there being no difference in the expression of 3NT, the biomarker for oxidative stress between treatment groups (Figure S2), the topical administration of DiOHF for 28 days significantly lowered collagen deposition at the bleb site in rabbits having undergone GFS when compared to the vehicle (Figure 2A). Collagen accumulation at the bleb was determined by picrosirius red staining. Densely packed, concentrated red collagen fibres were observed in the vehicle-treated bleb areas (Figure 2B), followed by less densely packed red fibres observed in MMC-treated blebs (Figure 2D), with DiOHF-treated blebs presenting with the least densely packed red collagen fibres (Figure 2C).

### 2.3. DiOHF Treatment Reduced Inflammation in Blebs More Effectively than the Vehicle or MMC Treatment in Rabbits with GFS

**Figure 3:**
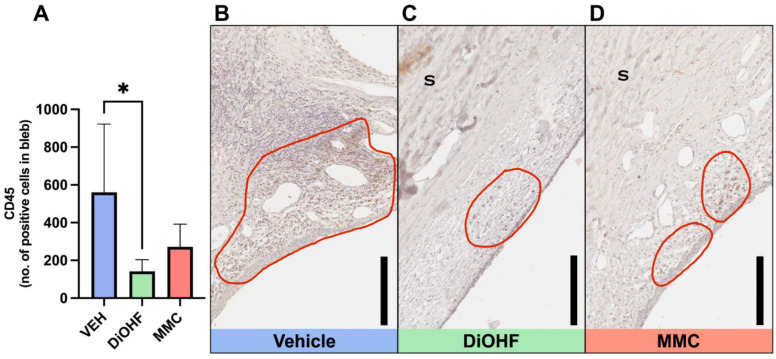
CD45 expression in blebs following 28 days post-GFS. (**A**) Quantitative data of total CD45-positive cells within the cross-sectional bleb region following post-GFS treatment with the vehicle, DiOHF or MMC. Vehicle, 0.01% DMSO; DiOHF, 10 μM; MMC, 0.4 mg/mL (One-way ANOVA with Tukey’s post hoc test, *n* = 5, * indicates a significance of *p* < 0.05. Data are presented as mean ± SD). (**B**–**D**) Representative images of blebs from operated rabbit eyes that received post-GFS treatment at the surgical site with either daily vehicle eye drops, daily DiOHF eye drops, or a one-time intraoperative application of MMC. Bleb areas containing CD45-positive cells are outlined in red. CD45-positive cells are stained brown, with nuclei counterstained with haematoxylin. S, sclera. Scale bar, 200 μm.

Clinical bleb analysis on days 7 and 28 revealed no change in inflammation for MMC-treated eyes; however, blebs in the vehicle and DiOHF treatment groups exhibited a decrease in inflammation over time (Figure 1B). Of the latter two groups, the DiOHF treatment group presented a more significant reduction in bleb inflammation from day 7 to day 28 (Figure 1B). Bleb inflammatory responses were likewise quantified by CD45-positive cell count following the immunohistochemical staining of tissue harvested at day 28 (Figure 3A). Treatment with DiOHF for 28 days significantly reduced intra-bleb CD45-positive cell count in comparison to the vehicle control (Figure 3A). DiOHF-treated blebs present with a smaller amount of brown-stained CD45-positive cells when compared to the vehicle- and MMC-treated blebs (Figure 3B–D).

### 2.4. Expression of CD31 in Post-GFS Rabbit Blebs Was Lowered with DiOHF Treatment

**Figure 4:**
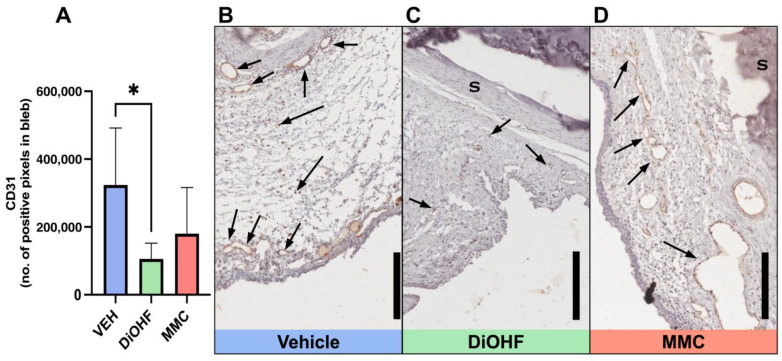
CD31 expression in rabbit blebs at 28 days following GFS. (**A**) Quantitative data, by positive pixel count, of total CD31-positive bleb areas following post-GFS treatment with the vehicle, DiOHF or MMC. Vehicle, 0.01% DMSO; DiOHF, 10 μM; MMC, 0.4 mg/mL (One-way ANOVA with Tukey’s post hoc test, *n* = 5, * indicates a significance of *p* < 0.05. Data are presented as mean ± SD). (**B**–**D**) Representative images of blebs from operated rabbit eyes that received post-GFS treatment at the surgical site with either daily vehicle eye drops, daily DiOHF eye drops, or a one-time intraoperative application of MMC. CD31-positive areas are stained brown and counterstained with haematoxylin. CD31-positive blood vessels are identified by arrows. S, sclera. Scale bar, 200 μm.

The immunohistological analysis of angiogenesis was assessed by the expression of the endothelial cell marker CD31 in rabbit bleb tissue at day 28. Daily topical administration of DiOHF reduced CD31 expression in conjunctival rabbit blebs compared to the vehicle-treated blebs (Figure 4A). Vehicle-treated blebs contain the largest amount of brown CD31-positive-stained blood vessels (Figure 4B), followed by a lesser amount observed in MMC-treated blebs (Figure 4D), with DiOHF-treated blebs presenting with the fewest and faintest brown CD31-positive-stained blood vessels (Figure 4C).

### 2.5. DiOHF and MMC Treatments Suppress Myofibroblast Expression in Rabbit Blebs Post-GFS

**Figure 5:**
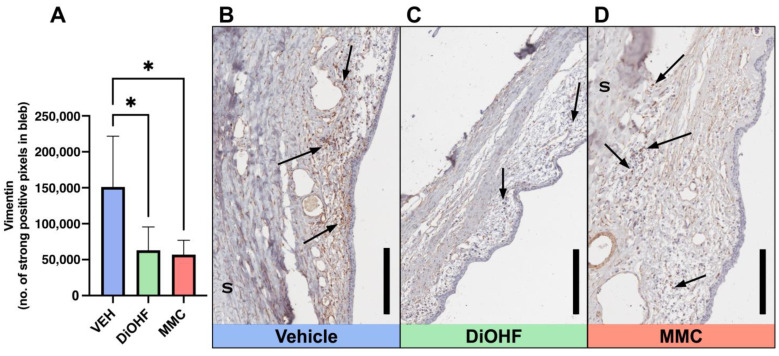
Expression of Vimentin in blebs at 28 days post-GFS. (**A**) Quantitative data, by strong positive pixel count, of total Vimentin-positive bleb areas following post-GFS treatment with the vehicle, DiOHF or MMC. Vehicle, 0.01% DMSO; DiOHF, 10 μM; MMC, 0.4 mg/mL (One-way ANOVA with Tukey’s post hoc test, *n* = 5, * indicates a significance of *p* < 0.05. Data are presented as mean ± SD). (**B**–**D**) Representative images of blebs from operated rabbit eyes that received post-GFS treatment at the surgical site with either daily vehicle eye drops, daily DiOHF eye drops, or a one-time intraoperative application of MMC. As indicated by black arrows, Vimentin-positive areas are stained brown, with nuclei counterstained with haematoxylin. S, sclera. Scale bar, 200 μm.

**Figure 6:**
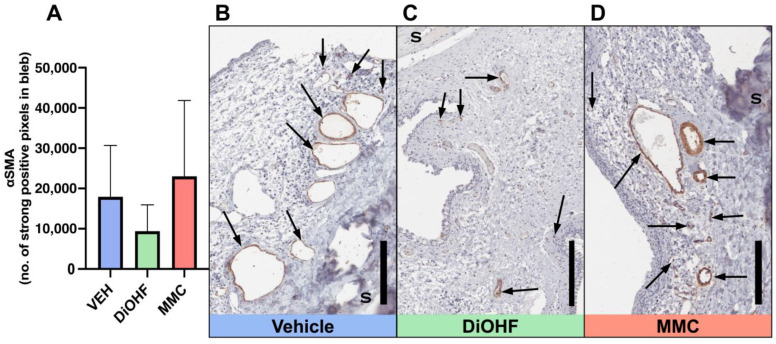
Expression of αSMA in GFS blebs after 28 days. (**A**) Quantitative data, by strong positive pixel count, of total αSMA-positive bleb areas following post-GFS treatment with the vehicle, DiOHF or MMC. Vehicle, 0.01% DMSO; DiOHF, 10 μM; MMC, 0.4 mg/mL (One-way ANOVA with Tukey’s post hoc test, *n* = 5). Data are presented as mean ± SD). (**B**–**D**) Representative images of blebs from operated rabbit eyes that received post-GFS treatment at the surgical site with either daily vehicle eye drops, daily DiOHF eye drops or a one-time intraoperative application of MMC. αSMA-positive areas are stained brown and counterstained with haematoxylin. αSMA-positive cells and blood vessels are indicated by arrows. S, sclera. Scale bar, 200 μm.

Myofibroblast expression at the bleb site was identified by conducting immunohistochemical staining on day 28 post-GFS rabbit bleb tissue for vimentin and alpha-smooth muscle actin (αSMA). Daily topical administration of DiOHF significantly suppressed vimentin expression at the bleb when compared to the vehicle treatment (Figure 5A). High levels of vimentin expression are observed in vehicle-treated blebs, as there are many brown-stained cells that occupy the bleb area (Figure 5B). Conversely, DiOHF- and MMC-treated blebs contain less vibrant and less concentrated brown staining, indicating minimal vimentin expression within the blebs (Figure 5C,D). We observe a trend of DiOHF-induced decrease in αSMA expression; however, no significant change was identified between treatment groups (Figure 6A). Yet, when DiOHF-treated tissue sections are observed alongside those treated with the vehicle or MMC, DiOHF-treated blebs contain the weakest and most dispersed brown-stained αSMA-positive cells (Figure 6B–D).

## 3. Discussion

The success of GFS currently remains limited by late-stage postoperative wound healing, characterised by excessive scar formation at the bleb. In an attempt to address this challenge, current clinical practice resorts to the use of potentially harmful antimetabolites, like MMC, as adjunctive antifibrotic therapy. While this approach can be effective, treatment with MMC is linked to postoperative complications such as wound breakdown and delayed hypotony, which can cause many vision-threatening pathologies [6,7,8,9,10]. As such, there is an unmet clinical need for a safer adjunctive antifibrotic therapy for filtering glaucoma surgeries, particularly trabeculectomy. Our study of the synthetic flavonol, DiOHF, as a superior alternative to MMC shows promise in resolving the current clinical dilemma. GFS-operated rabbit eyes treated postoperatively with DiOHF over the course of 28 days produced an overwhelmingly antifibrotic response demonstrated by a clear reduction in key markers of inflammation, angiogenesis, fibroblast accumulation and ECM remodelling.

In models of ocular fibrosis, pathological wound healing is typically characterised by fibrous scar formation in the later phases of wound healing. This scar formation is the result of an increased expression of fibroblasts in granular tissue and their transition into myofibroblasts during earlier phases of wound healing; GFS triggers an initial inflammatory response which recruits immune cells to the bleb site where they then secrete a host of cytokines, chemokines and growth factors including TGFβ. These secretions are known to stimulate fibroblasts, specifically, those localised to the conjunctiva and Tenon’s layer, to express their collagen-producing myofibroblastic phenotype [18]. In addition to increased production of extracellular matrix proteins like collagen, other key features which are characteristic of myofibroblast differentiation/activation include high levels of vimentin expression and αSMA expression [19]. In this study, we found that after 28 days, compared to the vehicle, DiOHF-treated rabbit blebs expressed reduced levels of vimentin and collagen deposition while also showing a trend toward a decrease in αSMA expression. The observed decrease in the expression of vimentin indicates reduced EMT and fewer fibroblasts accumulating at DiOHF-treated blebs during post-GFS wound healing. The reduction in vimentin expression is associated with decreased collagen deposition observed in DiOHF-treated blebs, since with there being fewer fibroblasts present in the bleb area, there are fewer fibroblasts available for activation into myofibroblasts, which then limits the amount of collagen that can be produced and deposited at the bleb site as a result.

While GFS is known to promote myofibroblast differentiation through TGFβ signalling, the propagation of TGFβ-mediated fibrogenesis is dependent on the production of mitochondrial reactive oxygen species (mtROS) by myofibroblasts in the early phase of wound healing [20,21]. By day 28, however, the wound healing process is considered to be at a late stage, which is characterised by the loss of the majority of αSMA-positive myofibroblasts at the surgical site [22,23,24]. We suspect that at this stage, we are likely observing the dedifferentiation of myofibroblasts to their quiescent fibroblastic form. Our reasoning for this derives from the comparison of vimentin and αSMA expression in bleb tissue of each treatment group after 28 days. Across all treatment groups, the mean number of αSMA-positive pixels was 60–88% lower than the mean number of vimentin-positive pixels detected at the bleb. This finding indicates that there was a greater number of fibroblasts accumulated in the bleb tissue than myofibroblasts. This would explain why, when compared to other treatment groups, our vehicle control group did not exhibit a significantly increased expression of 3NT or αSMA yet yielded significantly increased collagen deposition and thick fibrous scar formation at the bleb. Staining at day 28 with the marker 3NT likewise yielded non-specific background staining across all treatment groups. This further supports our theory of ROS production playing a pivotal role in the early, rather than later, stage of fibrosis.

During GFS, unavoidable incisions to the conjunctiva and sclera are perceived by the body as tissue damage. In response to this perceived injury, local immune cells such as leukocytes become activated and secrete proinflammatory cytokines that mediate local inflammation at the surgical site. Cytokines promote inflammation by influencing vascular permeability and endothelial cell interactions with inflammatory cells. In various chronic inflammatory and fibrotic diseases, oxidative stress resulting from upregulated mitochondrial and NOX-mediated ROS production has been recorded to stimulate various proinflammatory signalling pathways [25,26]. In this study, we found that treatment with the antioxidant DiOHF significantly reduced the accumulation of CD45-positive leukocytes at the bleb when compared to the vehicle control. This finding supports the literature on flavonoids inhibiting the infiltration of leukocytes [27]. These compounds are also known to possess strong antiangiogenic characteristics, which inhibit macrophages from producing angiogenic factors [28,29]. An important factor contributing to the surgical success of GFS is the degree of postoperative angiogenesis observed at the bleb. While a certain degree of angiogenesis is fundamental for supplying bleb tissue with nutrient-rich blood, an excessive amount of angiogenesis can promote a pathological proinflammatory and profibrotic outcome. In this way, our investigation of DiOHF was directed toward reducing, but not completely eliminating, the blood supply to the filtering bleb after surgery. Recent findings have revealed that treatment with flavonoids can influence multiple angiogenic factors and cascades in a dose-dependent manner such that the outcome can be either pro- or anti-angiogenic in nature [30,31]. This phenomenon was demonstrated both in vivo and in vitro with baicalin, which at low doses promoted angiogenesis and at high doses inhibited angiogenesis [32,33]. Following 28 postoperative days of treatment with DiOHF thrice daily, rabbit blebs exhibited significantly reduced levels of angiogenesis in comparison to blebs treated with the vehicle control. We suspect that this observed DiOHF-dependent reduction in bleb angiogenesis is associated with the DiOHF-dependent reduction in fibroblast accumulation, as fibroblasts have been found to secrete an array of soluble proteins which support endothelial cells in pro-angiogenic sprouting and lumen formation [34]. The antiangiogenic and anti-inflammatory effects of DiOHF hence contribute to the overall antifibrotic outcome observed in post-GFS rabbit eyes.

In this study’s evaluation of antifibrosis, treatment dosage and application were two distinct factors of consideration. Our selection of 10 μM as the applied dose of DiOHF was determined based on previous in vitro and murine studies of GFS, diabetes, ischaemia and reperfusion injury [15,18,35]. While our group’s recent study of GFS wound healing in mice demonstrated that daily intraperitoneal administration revealed the antifibrotic effect of DiOHF [18], this delivery route is not ideal for clinical translation. As such, we investigated the effect of topically administered DiOHF in eye drop form, as this would align with clinical standard-of-care medication delivery. The findings of this study confirm that the antifibrotic effect of DiOHF can be observed following GFS with daily topical application of eye drops containing DiOHF. Our chosen delivery of MMC is currently being performed clinically in GFS patients. That is, a one-time intra-bleb exposure of 0.4 mg/mL of MMC via cellulose sponge for 1 min followed by thorough irrigation with saline. While this method of application has been met with antifibrotic results clinically, in this study, we received an underwhelming overall response from treatment with MMC, with MMC-treated blebs exhibiting no significant difference in any of the IHC-tested parameters except for vimentin expression. We suspect that this lack of desired antifibrotic response is the result of either the concentration of MMC or the method of application not effectively penetrating the thick Tenon’s layer in rabbit eyes during GFS. The Tenon’s capsule is reported to be thicker in rabbits than in humans, and recent data have revealed that the younger an eye is, the thicker the Tenon’s capsule is [36]. As the majority of GFS patients are elderly, it would stand to reason that they would have a thinner Tenon’s capsule than rabbits that are 10–12 weeks old. In light of this, the following alternative MMC dose and application methods should be considered in order to more appropriately deliver MMC to a rabbit’s Tenon’s capsule. The first alternative would be to expose the surgical site to the 0.4 mg/mL MMC-soaked sponge for longer than 1 min. Studies in which MMC was applied to the rabbit bleb area for 5 min observed the intended response of MMC-treated tissue as a positive control [37,38]. The second alternative could be to apply MMC at a concentration exceeding 0.4 mg/mL for the same or shorter time; however, the literature rarely strays from, let alone exceeds, 0.4 mg/mL. Another possible alternative for optimising the delivery of MMC to the rabbit Tenon’s capsule would be to preference injecting a lower concentration of MMC directly into the Tenon’s rather than topically applying 0.4 mg/mL MMC diffusely to the bleb region via cellulose sponge. In their comparison of these two delivery methods, Swogger et al. determined that an intra-Tenon injection (0.2 mL × 0.1 mg/mL) of MMC was more effective than 0.4 mg/mL MMC applied via cellulose sponge for 4 min. The rabbit eyes treated with the sponge-applied MMC in Swogger et al.’s study showed a higher-than-expected level of collagen accumulation at the bleb [39], which aligns with the higher-than-expected level of collagen accumulation observed at MMC-treated bleb in this present study.

While the hypothesised antifibrotic effect of DiOHF was demonstrated, this study was limited in some ways. Firstly, the sample size for this study was considerably small, with only *n* = 5 per treatment group. Secondly, the experimental design of the MMC application did not allow for MMC to effectively penetrate the intended tissue in order to act as a positive control. This limited our ability to compare the efficacy of DiOHF as an antifibrotic against MMC. While we determined the optimal DiOHF dose of 10 μM from prior studies, similar preliminary tests for MMC dose and application could have been beneficial in ensuring MMC penetration rather than relying on current clinical practice. Thirdly, the majority of data analysed in this study was collected on Day 28, in the late stage of post-GFS wound healing. If we had a larger sample size, there might have been an opportunity to harvest eyes from each treatment group on Day 7 or Day 14 to analyse the effect of DiOHF on bleb tissue in the earlier stages of wound healing. Furthermore, aqueous humour samples were not collected in this study; therefore, we have no information about the activity of other inflammatory and anti-inflammatory cytokines present. In this study, we selected 3NT as our indicator of oxidative stress. Given that 3NT is a product of tyrosine nitration by free radicals, this may be too specific of a biomarker to determine the true extent of oxidative stress within the bleb. In future studies, it may be more appropriate to use various biomarkers of oxidative stress. Ideally, we would have liked to conduct this study in a larger animal model, like a primate, on the principle that humans and primates share a higher level of similarity in ocular anatomy than humans and rabbits. In the future, we hope to evaluate the antifibrotic effect of DiOHF in a larger animal model and then in a human clinical trial.

## 4. Materials and Methods

### 4.1. Surgical and Drug Treatment Procedure

Animal care was conducted in adherence to the ARVO Statement for the Use of Animals in Ophthalmic and Vision Research. All procedures were performed following approval from the institutional animal care and use committee (St Vincent’s Animal Ethics Committee; protocol no. 006/21). GFS was performed in the right eye of 15 New Zealand white female rabbits (10–12 weeks old; sourced from Piper Farm NSW and Flinders University, SA), with the fellow (left) eye serving as a non-surgical control. Ten minutes prior to surgery, the sedative acepromazine (0.5 mg/kg, Rimadyl^®^ solution ACP2, CEVA Animal Health, Sydney, NSW, Australia)) and analgesic carprofen (1.5 mg/kg, Zoetis, Kirkland, QC, Canada) were administered via subcutaneous injection to relax the rabbit. Following sedation, general anaesthesia, isoflurane (3–4% for induction, 1–2% for maintenance, Pharmachem, Brisbane, QLD, Australia) was delivered via inhalation. Local anaesthetic proparacaine hydrochloride solution (0.5%, Alcaine^®^, Alcon, Novartis Pharmaceuticals, Sydney, NSW, Australia) was administered topically to the eye undergoing surgery.

As per Figure S3A, rabbits were randomly assigned to receive treatment with (1) the vehicle (*n* = 5) or (2) DiOHF (10 μM; *n* = 5; Sigma-Aldrich, Castle Hill, NSW, Australia) following GFS, or (3) MMC (*n* = 5; Sigma-Aldrich, Castle Hill, NSW, Australia) during surgery. Artificial tears containing the vehicle (0.01% dimethyl sulfoxide (DMSO); Sigma-Aldrich) or DiOHF (10 μM; Sigma-Aldrich) were administered topically to both eyes thrice daily over 28 days (Figure S3B). MMC (0.4 mg/mL; Sigma-Aldrich) was applied intraoperatively to the subconjunctival space via a soaked cellulose sponge for 1 min prior to entry into the anterior chamber (Figure S3B). Thorough irrigation of the MMC-treated area was performed with 20 mL of 0.9% sodium chloride using a syringe. The surgical procedure involved dissection into the subconjunctival space and the creation of a drainage pathway connecting the anterior chamber and the subconjunctival space. Immediately following surgery, the eyes were imaged and topical antibiotic eye drops, chloramphenicol (0.5%, Chlorsig^®^, Aspen Pharmacare Australia, Sydeny, NSW, Australia), were applied to the operated eye to prevent postoperative infection. Additionally, topical corticosteroid dexamethasone sodium phosphate 0.1% (Maxidex^®^, Alcon, Novartis Pharmaceuticals, Sydney, NSW, Australia) was administered to the operated eye 1–3 times per day for 7 days after GFS (Figure S3A).

Operated eyes were photographed at days 0, 7, 15, 21 and 28 (Figure S1), while their blebs were scored (according to Table S1) for size, inflammation and ischaemia to provide a correlation of the clinical bleb characteristics with the histological analyses of fibrosis (Figure 1). At study termination on day 28, eyes were retrieved for biological and histological analyses.

### 4.2. Picrosirius Red Staining

Collagen accumulation at the bleb was detected by staining with picrosirius red (ab150681; Abcam, Melbourne, VIC, Australia) as per the manufacturer’s instructions and mounted in dibutyl phthalate polystyrene xylene (DPX; Labworks, Melbourne, VIC, Australia). Four distinct sections from each animal subject underwent staining with picrosirius red. Stained tissue was imaged with an Aperio Scanner (Aperio CS 2; Leica Biosystems, Mount Waverley, VIC, Australia). Picrosirius red–positive staining at the bleb sites was identified using a positive pixel count algorithm (Positive Pixel Count v9, Aperio ImageScope software; Leica Biosystems, Mount Waverley, VIC, Australia). The picrosirius red positive pixel counts were averaged from the four tissue sections collected.

### 4.3. Immunohistochemistry

Immunohistochemical (IHC) staining for αSMA, Vimentin, CD31, 3NT and CD45 was performed to identify markers of the wound healing response. Four distinct sections were collected per stain from each animal. Bleb sections underwent heat-mediated antigen retrieval in a citrate buffer (10 mM, pH 6.0 at 90 °C). Sections were then blocked with either Ultravision protein block (TA-125-PBQ; ThermoFisher Scientific, Fremont, CA, USA) or Dako protein block (X090930-2; Dako, Carpinteria, CA, USA). Sections were then incubated overnight at 4 °C after being stained with one of the following primary antibodies: CD45 antibody (1:200 dilution with PBS; MCA808GA; Bio-Rad, Hercules. CA, USA) to identify inflammatory cells; Vimentin antibody (1:200 dilution with PBS; M0725; Dako, Carpinteria, CA, USA) to identify fibroblasts; α-SMA antibody (1:200 dilution with PBS; M0851; Dako, Carpinteria, CA, USA) to identify smooth muscle actin in myofibroblasts; CD31 antibody (1:100 dilution with Dako EnVisionTM Flex Antibody Diluent (DM830); M0823; Dako, Carpinteria, CA, USA) as a marker of angiogenesis; 3NT antibody (1:100 dilution with PBS; ab7048; Abcam, Cambridge, CB2 0AX, UK) as a marker of oxidative stress, identifying the presence of ROS; and mouse IgG1 antibody (1:50 dilution with PBS; X0931, Dako, Carpinteria, CA, USA) as a negative control. Sections were incubated with HRP-conjugated anti-mouse secondary antibodies and visualised with 3,3′ -diaminobenzidine (DAB) chromogen (K3468; Dako, Carpinteria, CA, USA). Sections were counterstained with haematoxylin (Amber Scientific, Midvale, WA, Australia) and mounted in dibutyl phthalate polystyrene xylene (DPX; Labworks, Melbourne, VIC, Australia). Stained tissue was imaged with an Aperio Scanner (Aperio CS 2; Leica Biosystems, Mount Waverley, VIC, Australia). Positive staining at the bleb sites for αSMA, Vimentin, CD31, and 3NT was identified using a positive pixel count algorithm (Positive Pixel Count v9, Aperio ImageScope software; Leica Biosystems, Mount Waverley, VIC, Australia). Positive pixel counts were averaged from the four tissue sections collected. CD45-positive cells within the bleb area were manually counted and recorded using an Olympus BX61 microscope at 40× magnification, where CD45-positive cells were identified as cells having blue nuclei with a brown cell membrane.

### 4.4. Statistics

Data are expressed as the mean ± standard deviation (SD). Normality was assigned to data that passed the Shapiro-Wilk Test with *p* > 0.05. Where data were normally distributed, mean data were analysed with parametric one-way analysis of variance (ANOVA) followed by Tukey’s post hoc analysis using Prism 10.0 (GraphPad, San Diego, CA, USA). Statistical significance was attributed to a value of *p* < 0.05. Where data were not or could not be transformed to present as normally distributed, mean data were analysed with non-parametric Kruskal-Wallis ANOVA followed by Dunn’s post hoc analysis using Prism 10.0 (GraphPad, San Diego, CA, USA). Statistical significance of *p* < 0.05 is signified by * or #, while ## signifies *p* < 0.001.

## 5. Conclusions

In conclusion, our findings have demonstrated that the daily topical administration of DiOHF for 28 days promotes antifibrosis in a rabbit model of GFS. While we could not conclusively determine differences in intra-bleb oxidative stress levels between treatment groups, treatment with the antioxidant DiOHF exhibited antifibrosis through its anti-inflammatory, antiangiogenic and antifibroproliferative properties. Reduced inflammation was observed both in reduced redness of DiOHF blebs and decreased CD45 expression in DiOHF bleb tissue. The antiangiogenetic properties of DiOHF were observed via decreased CD31 expression in DiOHF blebs and reduced intra-bleb blood vessels. DiOHF demonstrated antifibroproliferative properties by reducing the expression of vimentin-positive fibroblasts, key effector cells of fibrosis. The combined effect of these properties of DiOHF resulted in the substantial reduction of collagenous scar tissue accumulation in DiOHF-treated blebs. While DiOHF treatment produced diffuse blebs that maintained a reduced yet appropriate amount of blood supply, MMC treatment produced ischaemic blebs. These findings indicate that DiOHF may be a safer and more effective wound-modulating agent than conventional antifibrotic therapies like MMC in GFS.

## Data Availability

The raw data for the bar charts will be made available by the authors on request.

## Supplementary Materials

The supporting information can be downloaded at https://www.mdpi.com/article/10.3390/ijms251910767/s1.
