# Supplementary material for: 3′,4′-Dihydroxyflavonol Inhibits Fibrotic Response in a Rabbit Model of Glaucoma Filtration Surgery
Source: Int J Mol Sci. 2024 Oct 7;25(19):10767. doi: 10.3390/ijms251910767 (PMC11476621; doi:10.3390/ijms251910767)
Supplement: Supplementary file 1 [file ijms-25-10767-s001.zip › ijms-3138773-supplementary.pdf]

International Journal of *Molecular Sciences*

MDPI

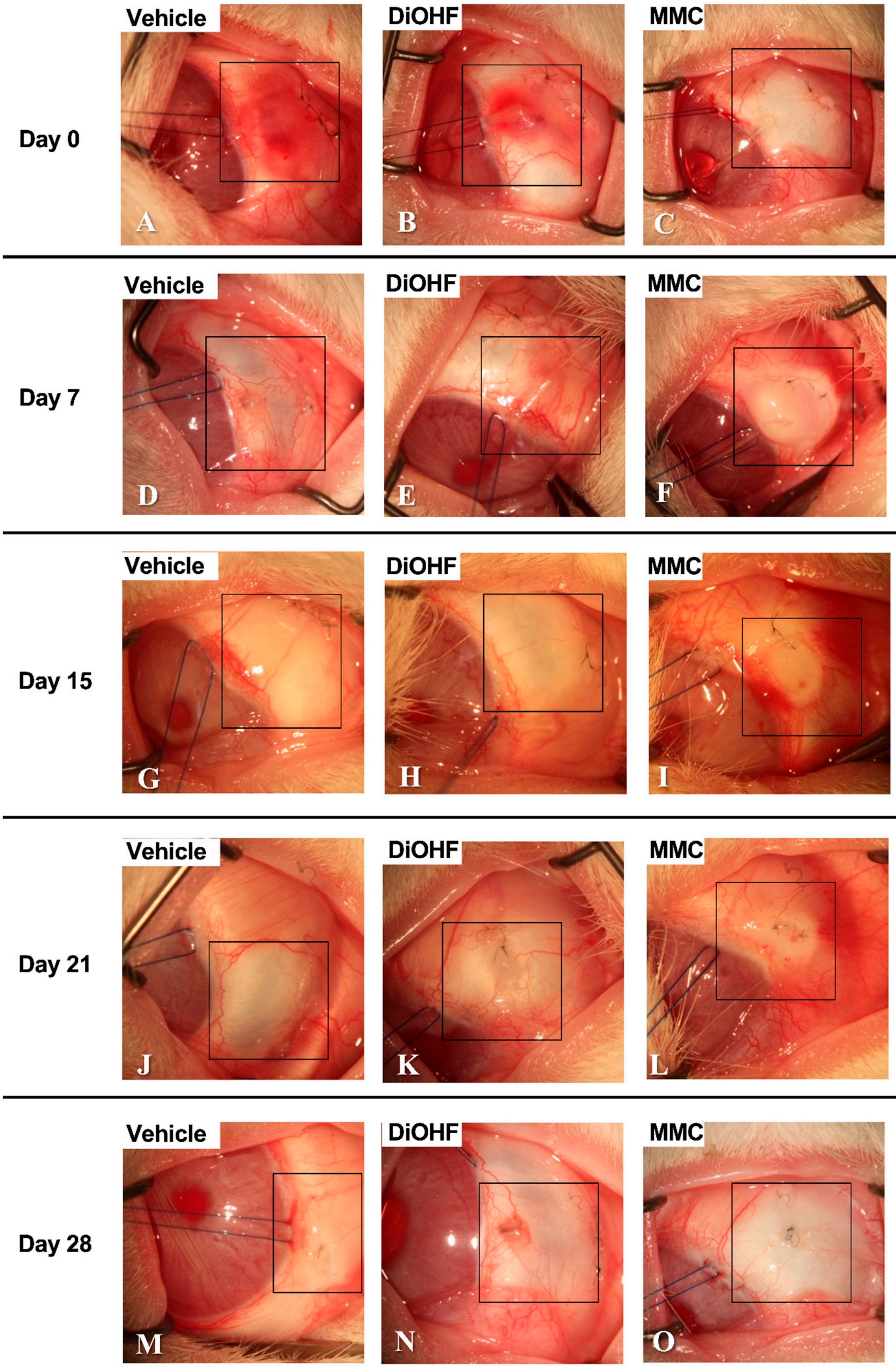

Supplementary Figure S1: Macroscopic images of rabbit filtering blebs following GFS. Images representative of each treatment group at ׀Day 0: (A) Vehicle, (B) DiOHF [10 uM] and (C) MMC [0.4 mg/mL]׀ ׀Day 7: (D) Vehicle, (E) DiOHF [10 uM] and (F) MMC [0.4 mg/mL]׀ ׀Day 15: (G) Vehicle, (H) DiOHF [10 uM] and (I) MMC [0.4 mg/mL]׀ ׀Day 21: (J) Vehicle, (K) DiOHF [10 uM]

and (L) MMC [0.4 mg/mL]| | Day 28: (M) Vehicle, (N) DiOHF [10 uM] and (O) MMC [0.4 mg/mL]. All blebs lie within the outlined region.

Supplementary Table S1. Grading Scale for Bleb Characteristics

	Size	Vascularity	Ischaemia scale
**0**	No Bleb	Not Present	Not Present
**1**	Small	Mild	Mild
**2**	Medium	Moderate	Moderate
**3**	Large	Severe	Severe

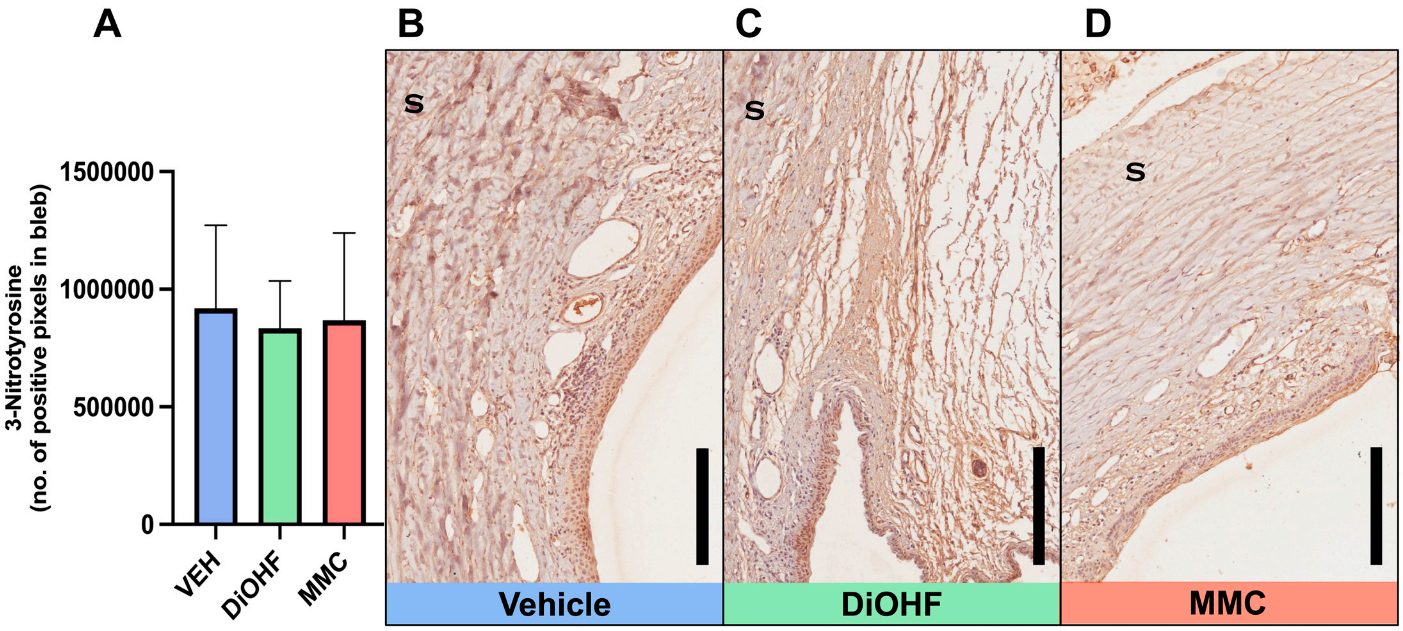

Supplementary Figure S2. 3NT expression in rabbit blebs at 28 days following GFS. (A) Quantitative data, by positive pixel count, of total 3NT-positive bleb areas following post-GFS treatment with vehicle, DiOHF or MMC. Vehicle, 0.01% DMSO; DiOHF, 10μM; MMC, 0.4 mg/mL (One-way ANOVA with Tukey's post hoc test, n=5, * indicates significance of P<0.05. Data are presented as mean ± SD). **(B-D)** Representative images of blebs from operated rabbit eyes that received post-GFS treatment to the surgical site with either daily vehicle eye drops, daily DiOHF eye drops or a one-time intraoperative application of MMC. S, sclera. Scale bar, 200μm.

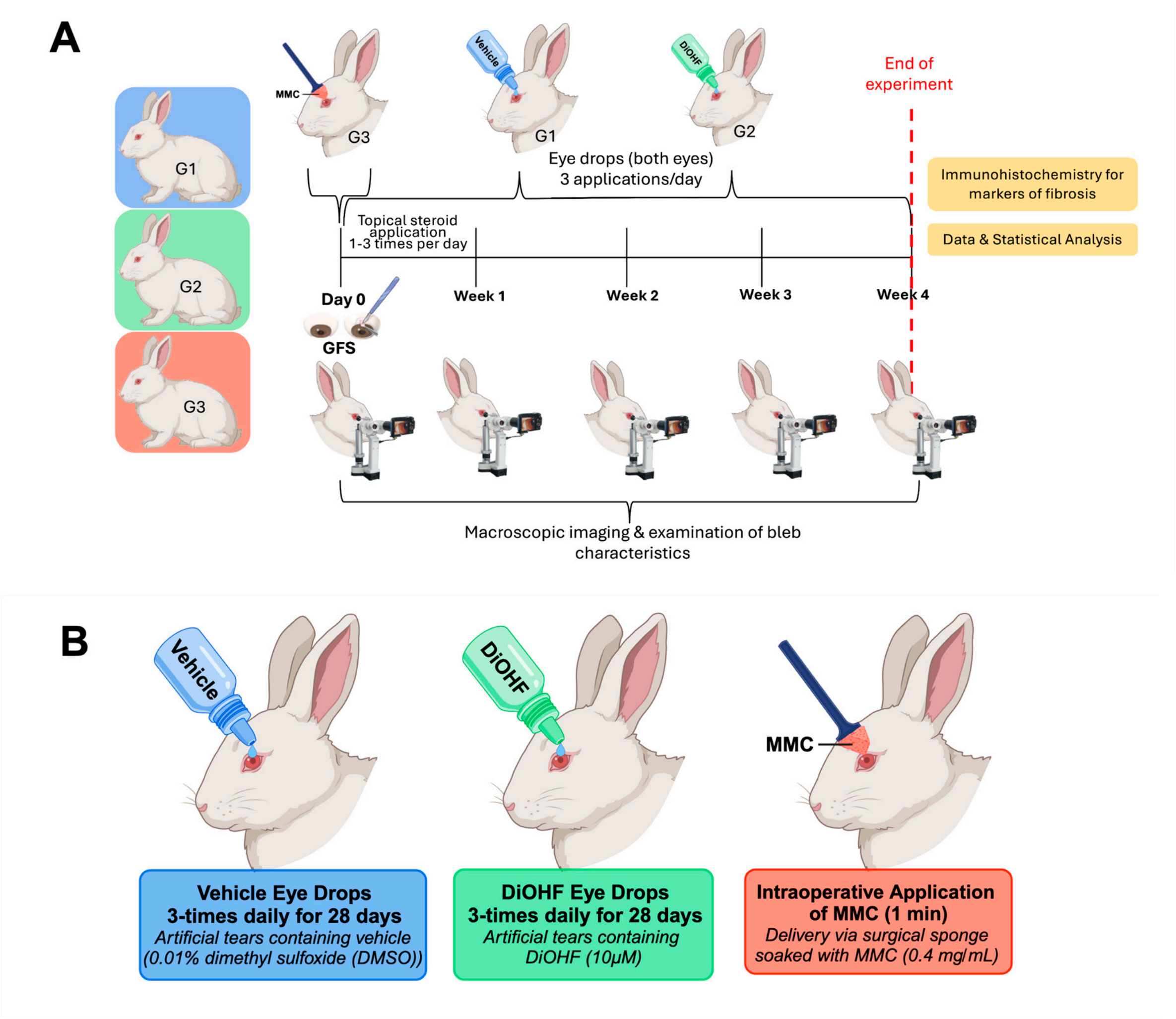

Supplementary Figure S3: Experimental Design. (A) Timeline of experiment over 4 weeks. **(B)** Detailed treatment groups. *Created with BioRender.com*

Disclaimer/Publisher's Note: The statements, opinions and data contained in all publications are solely those of the individual author(s) and contributor(s) and not of MDPI and/or the editor(s). MDPI and/or the editor(s) disclaim responsibility for any injury to people or property resulting from any ideas, methods, instructions or products referred to in the content.
